# Azole Resistance in Veterinary Clinical *Aspergillus fumigatus* Isolates in the Netherlands

**DOI:** 10.1007/s11046-024-00850-5

**Published:** 2024-06-12

**Authors:** Marloes A. M. van Dijk, Jochem B. Buil, Marlou Tehupeiory-Kooreman, Marian J. Broekhuizen, Els M. Broens, Jaap A. Wagenaar, Paul E. Verweij

**Affiliations:** 1 Faculty of Veterinary Medicine, Utrecht University, 3584 CL Utrecht, The Netherlands; 2 Department of Medical Microbiology, Radboud University Medical Center, 6525 GA Nijmegen, The Netherlands; 3 Wageningen Bioveterinary Research, 8221 RA Lelystad, The Netherlands; 4 Radboudumc-CWZ Center of Expertise for Mycology, 6525 GA Nijmegen, The Netherlands

**Keywords:** *Aspergillus fumigatus*, Aspergillosis, Azole resistance, Animals, Fungi

## Abstract

*Aspergillus fumigatus* is a saprophytic fungal pathogen that causes opportunistic infections in animals and humans. Azole resistance has been reported globally in human *A. fumigatus* isolates, but the prevalence of resistance in isolates from animals is largely unknown. A retrospective resistance surveillance study was performed using a collection of clinical *A. fumigatus* isolates from various animal species collected between 2015 and 2020. Agar-based azole resistance screening of all isolates was followed by in vitro antifungal susceptibility testing and *cyp51A* gene sequencing of the azole-resistant isolates. Over the 5 year period 16 (11.3%) of 142 *A. fumigatus* culture-positive animals harbored an azole-resistant isolate. Resistant isolates were found in birds (15%; 2/13), cats (21%; 6/28), dogs (8%; 6/75) and free-ranging harbor porpoise (33%; 2/6). Azole-resistance was *cyp51A* mediated in all isolates: 81.3% (T-67G/)TR_34_/L98H, 12.5% TR_46_/Y121F/T289A. In one azole-resistant *A. fumigatus* isolate a combination of C(-70)T/F46Y/C(intron7)T/C(intron66)T/M172V/E427K single-nucleotide polymorphisms in the *cyp51A* gene was found. Of the animals with an azole-resistant isolate and known azole exposure status 71.4% (10/14) were azole naive. Azole resistance in *A. fumigatus* isolates from animals in the Netherlands is present and predominantly *cyp51A* TR-mediated, supporting an environmental route of resistance selection. Our data supports the need to include veterinary isolates in resistance surveillance programs. Veterinarians should consider azole resistance as a reason for therapy failure when treating aspergillosis and consider resistance testing of relevant isolates.

## Introduction

*Aspergillus fumigatus* is a saprophytic fungal pathogen that causes opportunistic infections in animals and man, and can be isolated from soil [1, 2]. The inhalation of *A. fumigatus* conidia is the principal route of infection leading to pulmonary aspergillosis [2]. Hypersensitivity diseases such as allergic bronchopulmonary aspergillosis and severe asthma with fungal sensitization are common in humans. Invasive infections are less frequently seen and immunocompromised hosts are most at risk to develop such an infection [1, 3]. In both domesticated and wild animals *A. fumigatus* infection can have a variety of clinical manifestations, such as infection of the air sacs in birds, sino-nasal aspergillosis in dogs and cats, guttural pouch infection in horses and mycotic pneumoniae in ruminants and cetaceans [1, 4–6]. Similar to humans, immunocompromised animals, including animals kept in immuno-compromising conditions, e.g. poor husbandry, are more at risk to develop aspergillosis [1].

The treatment of choice for aspergillosis in human and veterinary medicine are azole antifungal drugs [1, 7], which inhibit ergosterol biosynthesis by binding the cytochrome P450 enzyme sterol 14α-demethylase (Cyp51) resulting in inhibition of fungal growth by cell membrane disruption [8, 9]. From the year 1998 onwards azole resistance has emerged in *A. fumigatus* [10, 11] and occurs worldwide with varying prevalence [12]. The ARTEMIS global surveillance study showed 5.8% azole resistance in *A. fumigatus* isolates [13] and a mainly European international surveillance network reported a prevalence of 3.2% [14]. The prevalence of azole-resistant *A. fumigatus* in humans in the Netherlands is 0.8–14.7% with TR_34_/L98H being the dominant resistance mechanism present in 69–94% of resistant isolates [10, 15–17]. Differences in the study period, isolate selection and geographical location will account for some of the difference in reported prevalence. However, it is clear that there is an increasing trend in resistance frequency [10, 16, 17]. Resistance has been demonstrated in clinical isolates from both azole treated and azole naive human patients [15]. The use of azole fungicides in agriculture is considered to lead to resistance selection in environmental *A. fumigatus* isolates [18–20]. In the Netherlands as many as 64.3% of patients with azole-resistant *Aspergillus* spp. were azole naive, supporting a plausible role of environmental resistance selection in human resistant *Aspergillus* disease [15].

Several azole resistance mechanisms have been described in *Aspergillus* spp. and for *A. fumigatus* the most important one is decreased azole affinity through amino acid substitutions within the Cyp51A target protein. These alterations are the result of single nucleotide polymorphisms (SNPs) in the encoding *cyp51A* gene [8, 9]. The most common SNPs associated with patient treatment with azole drugs are G54, G138, M220 and G448. In case of environmental exposure SNPs are accompanied by tandem repeats (TR) of base pairs in the promotor region of the *cyp51A* gene, leading to increased gene expression, with predominantly TR_34_/L98H and TR_46_/Y121F/T289A being found [21].

Few studies have been conducted to evaluate azole resistance in *A. fumigatus* isolates from animal infections. Most studies focus on birds as *A. fumigatus* is an important pathogen of the avian respiratory tract [22, 23]. Triazole resistance frequencies of 0–21% in poultry [24–27] and 0–17.1% in other birds [28–33] have been reported. Studies on *A. fumigatus* isolates from dogs and cats are very limited and only one triazole resistant Australian canine isolate is described so far [34]. This isolate harbored the non-synonymous F46Y and E427K mutations in the *cyp51A* gene [34], which are associated with reduced triazole susceptibility [35, 36].

The aim of this retrospective surveillance study was to determine the azole resistance frequency, resistance phenotype and genotype in a collection of clinical *A. fumigatus* isolates from various animal species collected over a period of 5 years.

## Materials and Methods

### Isolates

Between January 2015 and October 2020 all *A. fumigatus* isolates cultured from clinical samples submitted to the Veterinary Microbiological Diagnostic Center (VMDC) of the Faculty of Veterinary Medicine, Utrecht University, The Netherlands were stored, yielding 142 isolates. The VMDC receives samples from various animal species submitted by Dutch veterinarians and by the Pathology Department and Dutch Wildlife Health Centre (both part of Faculty of Veterinary Medicine). Samples submitted by the latter two were taken post mortem (n = 15). Samples were cultured on Sabouraud dextrose agar and malt extract agar (Biotrading, Mijdrecht, the Netherlands). Species identification was based on macroscopic and microscopic morphologies. If *A. fumigatus* colonies were present one random colony was subcultured on Sabouraud dextrose agar and stored in the biobank. Only one isolate was included for each animal. Information regarding geographical origin, i.e. owner postal code or location where the animal was found for wildlife, age, sex, clinical signs, prior azole treatment, therapy and clinical response were obtained from the submission form when provided. For all samples with an azole-resistant isolate submitting veterinarians/pathologists were contacted to obtain missing information.

### Antifungal Susceptibility Testing

The stored isolates were retrieved from the VMDC biobank and cultured on Sabouraud dextrose agar at 30 °C until sporulation occurred. According to the manufacturer’s instructions a 2 McFarland spore suspension was prepared in distillated water and a single drop (about 25 µl) was used to inoculate a commercial agar-based screening system to screen for azole resistance (VIPcheck™, https://www.vipcheck.nl). The system consists of a 4-well plate with 4 mg/l itraconazole (ITC), 2 mg/l voriconazole (VRC), 0.5 mg/l posaconazole (POS) and a growth control [37–39]. Plates were incubated at 37 °C for 48 h and checked for growth on day one and two of incubation. Antifungal susceptibility testing (AST) according to the EUCAST broth microdilution reference method (E.Def 9.3.2) was performed for isolates showing (minimal) growth on ≥ 1 azole-containing well. Minimal Inhibitory Concentrations (MICs) were determined for amphotericin B (AMB) and triazoles ITC, VRC, POS and isavuconazole (ISC). In addition MICs were determined for the imidazole miconazole (MCZ) for the aforementioned isolates and an additional random selection of four to five wildtype *A. fumigatus* veterinary isolates per year. In absence of veterinary breakpoints, EUCAST breakpoints for humans were used [40] for interpretation of MICs. Clinical breakpoints classify isolates as drug susceptible or resistant. For triazoles area of technical uncertainty (ATU) is defined for drug-bug combinations where there is uncertainty regarding the classification as susceptible or resistant. Clinical breakpoints were available for AMB (S ≤ /R >  = 1/1), ITC (S ≤ /R >  = 1/1, ATU = 2), VRC (S ≤ /R >  = 1/1, ATU = 2), POS (S ≤ /R >  = 0.125/0.25, ATU = 0.25) and ISC (S ≤ /R >  = 1/2, ATU = 2). Since there are currently no breakpoints available for MCZ, the epidemiological cutoff (ECOFF) was determined based on the wildtype MIC distribution [41]. Isolates were defined as azole-resistant if they were resistant to one or more triazole drug.

### Cyp51A Gene Sequencing

From isolates with an azole-resistant phenotype the *cyp51A* gene and promoter region were subsequently sequenced as described before [42].

### Statistical Analysis

Statistical analysis was performed using R version 4.2.3 [43]. For each year the azole-resistant proportion of isolates was calculated. A pairwise comparison of proportions using the Fisher’s exact test was used to determine whether differences between proportions were statistically significant (*p* ≤ 0.05). Figures were created using postal codes and publicly available data using datawrapper (https://www.datawrapper.de).

## Results

**Fig. 1:**
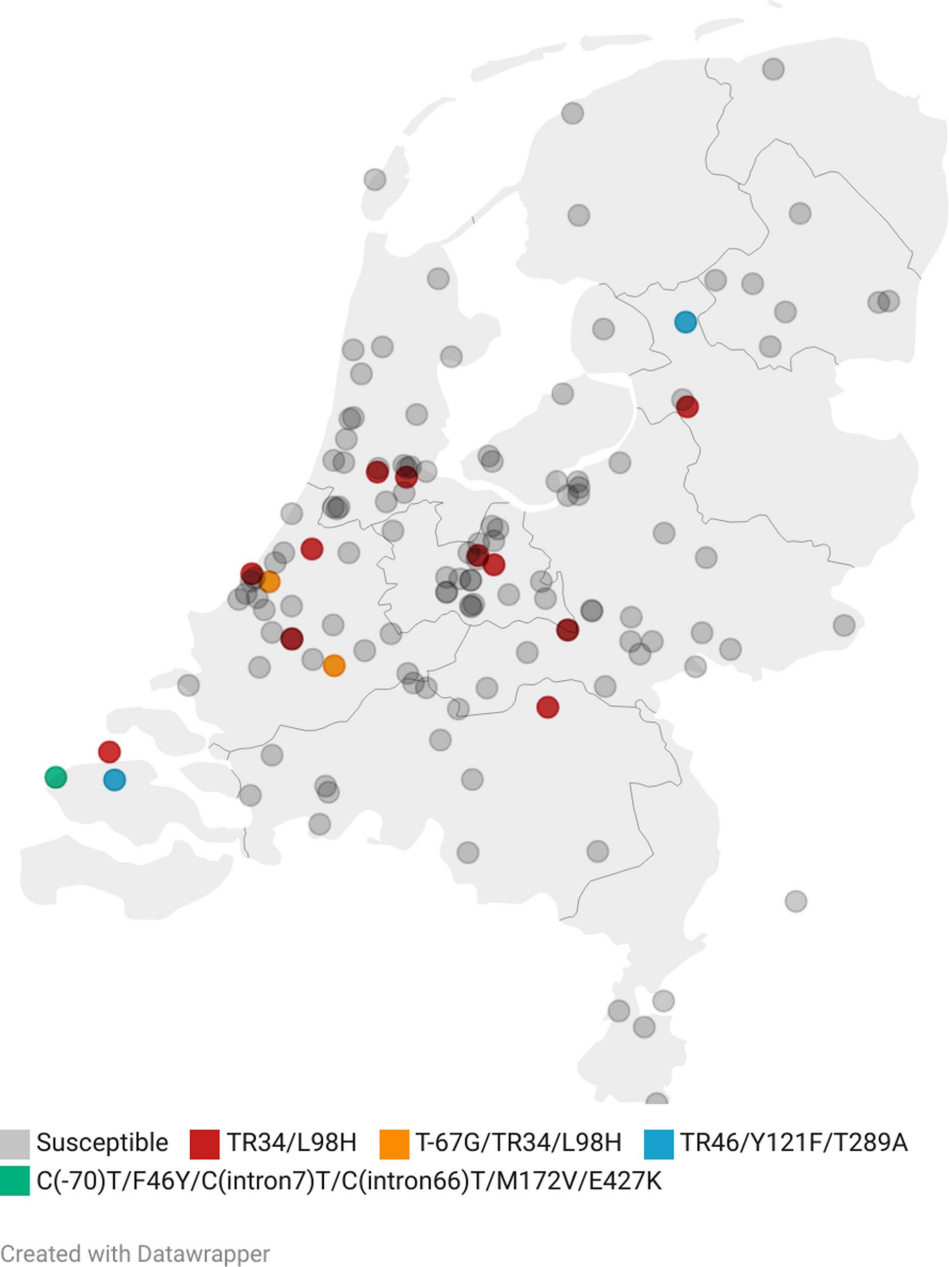
Geographical origin of *A. fumigatus* isolates and their resistance genotype. Created by datawrapper (https://www.datawrapper.de)

Isolates were cultured from different animal species and sites of infection (Table 1), originating from both wildlife (bird, hare and harbor porpoise) and kept animals (bird, cat, dairy cow, dog, gazelle, horse). The number of collected isolates per year, results of resistance screening and resistance frequencies are summarized in Table 2. Resistance screening identified 16 isolates showing growth on azole-containing wells. MIC-testing confirmed azole resistance in all 16 isolates, with a pan-azole-resistant phenotype in 15 (94%) isolates. One isolate (#12; cat, nose) showed multi-azole resistance for VRC, POS and ISC, but was susceptible to ITC. Azole resistance was *cyp51A* mediated in all sixteen isolates. TR_34_/L98H was the most prevalent resistance genotype with 81.3% (13/16); two isolates within this group harbored an additional T-67G substitution in the *cyp51A* promoter region. TR_46_/Y121F/T289A was found in 2 isolates (12.5%). In one isolate (#16; harbor porpoise, lung) a combination of C(-70)T/F46Y/C(intron7)T/C(intron66)T/M172V/E427K single-nucleotide polymorphisms in the *cyp51A* gene was found. The geographical origin of isolates and resistance genotype is shown in Fig. 1. Isolate distribution reflects population density [44] and no foci are seen.

**Table 1:** Origin of *Aspergillus fumigatus* isolates

Animal species	Sample origin	Number of samples
Bird^a^	Air sac	5
	Brain	1
	Choana	2
	Lung	1
	Nose	3
	Trachea	1
Cat	Bronchi	1
	External ear canal	18
	Middle ear	1
	Nose	7
	Sinus	1
Dairy cow	Lung	1
	Trachea	1
Dog	Blood	1
	Bronchi	2
	Conjunctiva	2
	External ear canal	15
	Heart	1
	Joint	3
	Lung	1
	Nose	48
	Sinus	2
Gazelle	Lung	1
Hare	Lung	1
Horse	Conjunctiva	8
	Nose	1
	Sinus	2
	Trachea	2
	Uterus	3
Harbor porpoise	Ear	2
	Lung	4
	Total	142

^a^Seagull, parakeet, ibis, duck, penguin, cormorant, parrot, swan

**Table 2:** Number and proportion of resistant isolates per year

Sampling year	Isolates collected (n)	Growth on azole-containing wells (VIPcheck™) (n)	Resistance frequency (%)	95% Confidence interval
2015	21	2	9.5	1.7–31.8
2016	25	1	4.0	0.2–22.3
2017	25	6	24.0	10.2–45.5
2018	21	1	4.8	0.2–25.9
2019	27	0	0	0.0–15.5
2020	23	6	26.1	11.1–48.7
Total	142	16	11.3	6.8–17.9

MICs for MCZ ranged from 2 to 8 mg/l (median 4 mg/l) for wildtype isolates (n = 29), resulting in a MCZ ECOFF of ≤ 8 mg/l. MCZ showed no activity against TR_34_-mediated resistant *A. fumigatus* isolates (MIC > 16 mg/l), whereas the MCZ MIC of two isolates harboring TR_46_/Y121F/T289A was 8 mg/l. The isolate harboring C(-70)T/F46Y/C(intron7)T/C(intron66)T/M172V/E427K showed a MCZ MIC of 16 mg/l. Azole resistance frequencies were elevated in the years 2017 and 2020, without a trend over the years.

Resistant isolates were found in birds, cats, dogs, and harbor porpoise. Characteristics of the sample population for these animal species and prior azole treatment status are shown in Table 3. For prior azole treatment status only the information provided in the submission form was used. Additional information obtained for samples with an azole-resistant isolate by contact with the submitting veterinarian/pathologist was not taken into account here to avoid bias. When stratified for animal species overall resistance frequencies were 15% (2/13) for birds, 21% (6/28) for cats, 8% (6/75) for dogs and 33% (2/6) for harbor porpoises, without significant differences (*p* > 0.05) between these proportions. Both birds with an azole-resistant isolate were captive birds, with two wildlife and nine captive birds in the group with azole-susceptible isolates. Overall and within species there were no significant differences (*p* > 0.05) in proportions of animals treated with azole antifungal drugs when comparing the azole-susceptible to the azole-resistant group.

**Table 3:** Sex, age and prior azole treatment for resistant and susceptible isolates per animal species

Animal species	Isolate R/S*	Number of isolates	Sex^**a**^			Median age (years)	Prior azole treatment (%)^**b**^
			M (n)	F (n)	Unknown (n)		
Bird	S	11	5	2	4	17.0	1 (9.1)
	R	2	1	0	1	1.5	1 (50)
Cat	S	22	8	13	1	11.0	4 (18.2)
	R	6	2	4	0	10.2	0
Dog	S	69	37	30	2	6.1	1 (1.4)
	R	6	5	1	0	5.9	1 (16.7)
Harbor porpoise	S	4	0	0	4	Unknown	0
	R	2	0	0	2	Unknown	0

*Isolates azole-resistant (R) or -susceptible (S) based on VIPcheck™ resistance screening

^**a**^M Male, F Female, Unknown: sex not stated in the submission form

^**b**^Based on information provided in the submission form

Azole-resistant isolates were cultured from the respiratory tract (n = 9) and the external ear canal (n = 7). Information on prior azole exposure, treatment and clinical outcome was acquired for fourteen of sixteen animals harboring an azole-resistant *A. fumigatus* isolate (Table 4); four animals (28.6%) received azole treatment within 1 week to 4 months prior to sampling and ten (71.4%) were azole naive. Prior treatment consisted of one to multiple courses with a duration of one to several weeks with MCZ or ITC. Six of the fourteen animals did not receive subsequent antifungal therapy, one was treated with ketoconazole, two with itraconazole, in one animal therapy information was unavailable and in four animals not applicable as samples were taken at pathological examination. Information on clinical outcome reported recovery for three of fourteen animals, four animals had died prior to sampling and no follow up information was available for seven animals. Death was attributable to aspergillosis in two of the four animals sampled at pathological examination (#6, #9) and for two animals this was unclear from the information provided by the pathologist.

**Table 4:** Sample origin and antifungal susceptibility results of azole-resistant isolates

	Sample origin								MIC in mg/l (interpretation^a^)						
Nr.	Sampling year	Animal species	Sampling site	Sex^b^	Age (year)	Prior azole treatment^c^	Antifungal therapy	Clinical outcome	AMB	ITC	VRC	POS	ISC	MCZ	*cyp51A* gene sequencing
1	2015	Dog	Ear	M	5	Unknown	Unknown	Unknown	0.5 (S)	> 16 (R)	4 (R)	0.5 (R)	4 (R)	> 16	TR_34_/L98H
2	2015	Cat	Ear	F	5	No	None	No follow up	0.5 (S)	> 16 (R)	8 (R)	0.5 (R)	4 (R)	> 16	TR_34_/L98H
3	2016	Cat	Ear	F	8	No	Ketoconazole	Recovered	0.5 (S)	> 16 (R)	4 (R)	0.25 (R)	4 (R)	> 16	T-67G/TR_34_/L98H
4	2017	Dog	Nose	F	6	No	None	No follow up	1 (S)	> 16 (R)	8 (R)	0.5 (R)	8 (R)	> 16	TR_34_/L98H
5	2017	Cat	Nose	M	15	No	None	Recovered	1 (S)	2 (R)^d^	> 16 (R)	1 (R)	> 16 (R)	8	TR_46_/Y121F/T289A
6	2017	Bird	Air sac	–	1	No	NA	Death^f^	0.5 (S)	> 16 (R)	8 (R)	0.5 (R)	8 (R)	> 16	TR_34_/L98H
7	2017	Cat	Ear	F	12	No	None	No follow up	0.5 (S)	> 16 (R)	4 (R)	0.5 (R)	4 (R)	> 16	TR_34_/L98H
8	2017	Dog	Ear	M	5	Miconazole	Unknown	No follow up	0.25 (S)	> 16 (R)	2 (R)^e^	0.25 (R)	4 (R)	> 16	T-67G/TR_34_/L98H
9	2017	Bird	Air sac	M	–	Itraconazole	NA	Death^f^	0.5 (S)	> 16 (R)	8 (R)	0.5 (R)	4 (R)	> 16	TR_34_/L98H
10	2018	Dog	Nose	M	11	No	Itraconazole	No follow up	0.25 (S)	> 16 (R)	4 (R)	0.5 (R)	8 (R)	> 16	TR_34_/L98H
11	2020	Dog	Ear	M	4	Miconazole	None	Recovered	1 (S)	> 16 (R)	8 (R)	1 (R)	16 (R)	> 16	TR_34_/L98H
12	2020	Cat	Nose	F	13	No	Itraconazole	No follow up	0.5 (S)	0.5 (S)	> 16 (R)	0.5 (R)	> 16 (R)	8	TR_46_/Y121F/T289A
13	2020	Cat	Ear	M	6	Miconazole	None	No follow up	0.25 (S)	> 16 (R)	8 (R)	0.5 (R)	8 (R)	> 16	TR_34_/L98H
14	2020	Dog	Nose	M	12	Unknown	Unknown	Unknown	0.5 (S)	> 16 (R)	8 (R)	0.5 (R)	8 (R)	> 16	TR_34_/L98H
15	2020	Harbor porpoise	Lung	–	–	No	NA	Death^f^	0.25 (S)	> 16 (R)	2 (R)^e^	0.25 (R)	2 (R)	> 16	TR_34_/L98H
16	2020	Harbor porpoise	Lung	–	–	No	NA	Death^f^	0.5 (S)	> 16 (R)	4 (R)	1 (R)	2 (R)	16	C(-70)T /F46Y/C(intron7)T/C(intron66)T/M172V/ E427K

^a^According to EUCAST [45]; S susceptible, R resistant; no clinical breakpoints available for MCZ

^b^M male, F female,- unknown

^c^based on information on the submission form complemented with information procured by contact with the submitting veterinarian/pathologist

^d^In some clinical situations (non-invasive infections forms) itraconazole can be used provided sufficient exposure is ensured

^e^In some clinical situations (non-invasive infections forms) voriconazole can be used provided sufficient exposure is ensured

^f^Death prior to sampling. Sample taken at pathological examination

## Discussion

In this study of *A. fumigatus* isolates from animals in the Netherlands an overall azole resistance prevalence of 11.3% was found. A Dutch national surveillance program is in place to monitor the prevalence of azole resistance in *A. fumigatus* in the human population. In this surveillance program five University Medical Centers and five teaching hospitals screen clinical *A. fumigatus* isolates using VIPcheck™, which corresponds to the methods used in our study. Human surveillance shows overall resistance rates of 10.7%, 12.9%, 14.7%, 10.5%, 9.1% and 8.2% for the years 2015–2020 respectively [46–51]. In addition to this surveillance program several studies have investigated the prevalence of azole resistance in clinical *A. fumigatus* isolates from the Dutch human population. One study from a tertiary care hospital found an overall patient azole-resistance prevalence of 5.3% in 2,051 patients in 1994–2016. Patient resistance frequency increased from 0% in 1997 to 9.5% in 2016 [17]. Two studies on isolates from multiple University Medical Centers found an overall patient azole-resistance prevalence of 5.3% in 1,192 patients in 2007–2009 [15] and 11% in 4,496 patients in 2013–2018, with annual resistance frequencies increasing from 7.6 to 14.7% [16]. Our study period and prevalence of 11.3% is in line with these findings from the Dutch human population. However, in our study no screening method was used at initial culture and only one random *A. fumigatus* isolate was stored. As mixed (azole-susceptible and azole-resistant) genotypes may be present in clinical cultures, azole-resistant colonies may have been missed [52] and true prevalence might be higher. In our study prevalence of resistance varied widely between the years and no trend was visible, which may be due to the limited number of *A. fumigatus* isolates per year.

Few studies have been conducted to evaluate azole resistance in *A. fumigatus* isolates from animals. We found a resistance frequency of 15% (2/13) in wild life and captive birds. Previous studies found azole-resistance in *A. fumigatus* isolates from four of 54 birds (7.4%) from Belgium and the Netherlands [29] and only azole susceptible isolates from nine birds from Japan [28]. It is difficult to interpret the outcomes of these studies, because sample background information is missing. A recent study reported azole resistance in 17.1% of clinical (6/35) and 11.8% (8/68) of environmental *A. fumigatus* isolates from Humboldt Penguins and other birds and their surroundings from 2017 to 2022 in a Belgian Zoo [32]. In this study sequencing of the *cyp51A* gene revealed the TR_34_/L98H mutation in all environmental isolates and TR_34_/L98H (2/6), F46Y/M172V/E427K (1/6) and the absence of known resistance mutations (3/6) in clinical isolates. Genotyping showed an interesting cluster of azole-resistant isolates from the same time point encompassing environmental strains and one clinical strain from a Humboldt pinguin indicating environment to bird transmission. Resistance frequency and mutations are in line with the results of our study and support the environment to animal transmission. Another study investigated 159 cases of aspergillosis in captive birds from Germany in 2015–2018 and found one (0.6%) azole-resistant *A. fumigatus* isolate [30]. In this study samples were collected from all avian aspergillosis cases seen at the University Clinic during the study period. In our study results are possibly biased as it is likely that samples were more often submitted from animals with severe disease and/or not responding to antifungal therapy, thus leading to a higher resistance frequency. Such differences in sample selection can lead to variation in azole-resistance prevalence.

Little is known of the prevalence of azole-resistant *A. fumigatus* in companion animals. In the Netherlands triazole resistance was not observed in 27 isolates from eight dogs with sino-nasal aspergillosis in 2010–2012 [53]. A study on 50 isolates originating from Australia, United States and Belgium collected between 1988 and 2014 from 46 dogs and four cats with sino-nasal aspergillosis showed one triazole resistant Australian canine isolate from 1992 [34]. Resistance frequencies for dogs (8%) and cats (21%) are much higher in our study. This might be due to a higher number of isolates, more recent study period and the possible submission bias in our study as suggested above. However, both studies might be prone to a similar bias as isolates were retrieved from a culture collection of a clinical research group on infectious diseases [34] or included dogs from a veterinary referral hospital only [53].

Studies investigating aspergillosis in harbor porpoises are scarce [5, 6] and none have investigated azole-resistance in *A. fumigatus* isolates. In our study two of the six *A. fumigatus* isolates from stranded, free-ranging harbor porpoises were azole-resistant. Both animals were deceased at stranding and suffered from fungal pneumonia, which has been described in harbor porpoise [5, 6].

Overall VIPcheck™ resistance screening accurately predicted azole-resistance in isolates showing (minimal) growth on ≥ 1 azole-containing wells, so specificity for this screening method is high. Isolates that only grew in the control well were not further tested, therefore sensitivity cannot be evaluated with our data. It is unlikely that many azole resistant isolates were missed, as studies evaluating the VIPcheck™ system have reported an excellent sensitivity and specificity of 98–99% and 93–99%, respectively [37, 54]. However, isolate number 15 underlines the importance of including minimal growth as a positive result, as it showed only minimal growth for ITC and VRC, while MIC-testing showed resistance to ITC, VRC and POS. This is in line with previous findings [37, 54] and underscores the necessity of subsequent MIC-testing for accurate antifungal susceptibility profiling.

Although triazole resistance has been broadly investigated in *A. fumigatus*, there is a paucity of in vitro susceptibility information on imidazole drugs. We included MCZ in our current study because imidazole drugs, e.g. MCZ, enilconazole, clotrimazole and ketoconazole, are licensed for use in animals [55] and some imidazole drugs are used for treatment of sino-nasal aspergillosis in dogs and cats [56, 57]. In human medicine MCZ is not considered an appropriate drug for treatment of *Aspergillus* diseases, due to various reasons including relatively high MIC-values [58]. We indeed found relatively high MICs in our wildtype *A. fumigatus* isolates with an ECOFF of ≤ 8 mg/l. This MIC distribution is very similar to that observed in an early study investigating the in vitro activity of MCZ [59]. MCZ showed no in vitro activity against TR_34_/L98H, whereas the MICs of two TR_46_/Y121F/T289A were at the ECOFF concentration. Studies with an imidazole fungicide, imazalil, indicated that the presence of a F495I SNP was associated with imidazole resistance [60]. Indeed MCZ showed no activity against two clinical *A. fumigatus* isolates harboring TR_34_/L98H/S297T/F495I [61]. However, our data suggest that MCZ also shows no activity against TR_34_/L98H sensu stricto isolates. Likely various factors including molecule structure and chemical characteristics and resistance mutation variations determine the resistance phenotype, underscoring the complexity of resistance in *A. fumigatus*.

The distribution of resistance genotypes was similar to that observed in the national resistance surveillance program, with dominance of TR_34_/L98H. In humans, in host resistance selection during triazole therapy is characterized by single resistance mutations mainly in the *cyp51A* gene. Such resistance mechanisms were not observed in veterinary isolates, possibly pointing towards a low risk of resistance selection during antifungal therapy. Prolonged azole therapy is less common in veterinary medicine and (to our knowledge) was not administered to animals in our study. However, often no information on previous azole therapy was provided and only actively obtained for samples with an azole-resistant isolate. It is therefore difficult to accurately assess the effect of previous azole exposure on our collection of isolates. In our study 71.4% of animals with an azole-resistant *A. fumigatus* isolate and known azole exposure status were azole naive, which is in line with the 64.3% previously found in the Dutch human population (2007–2009) [15] and indicates an environmental route of resistance development.

Resistance mutations (T67G/)TR_34_/L98H and TR_46_/Y121F/T289A are associated with environmental resistance selection [16, 21] and accounted for all but one (94%) mutations in our collection from animals. The mutations in the C(-70)T/F46Y/C(intron7)T/C(intron66)T/M172V/E427K isolate recovered from a free ranging harbor porpoise, have been described for both human clinical and environmental strains [35, 62]. This suggests that, similar to humans, the environmental resistance route is dominant in veterinary medicine and that veterinary azole use is an unlikely hotspot for resistance selection [19, 20]. Recent genomic studies confirm environment-to-patient transmission of azole-resistant *A. fumigatus*, with closely-related genomes of environmental and human TR_34_/L98H isolates [63]. Studies investigating the genomic epidemiology of resistance in animal isolates are lacking but will be required to confirm environment-to-animal transmission.

Due to the limited number of animals with an azole-resistant *A. fumigatus* isolate and paucity of clinical information it is impossible to make general statements regarding clinical implications of azole resistance for the animals in our study. However, the results of our study have implications for veterinary microbiological diagnostic laboratories and veterinarians. An expert panel made recommendations for resistance management depending on the resistance prevalence [64]. In an area with a local environmental resistance rate of < 5% screening for azole resistance was recommended for patients with insufficient clinical response to azole therapy, 5% was recommended as threshold for routine resistance screening of *A. fumigatus* isolates and 10% for reconsidering the use of azole monotherapy as primary treatment for patients with aspergillosis [21, 64]. In our study an overall azole resistance prevalence of 11.3% was found in clinical *A. fumigatus* isolates from animal infections. Therefore veterinarians should be aware of azole therapy failure due to resistance and diagnostic laboratories should consider offering screening of *A. fumigatus* isolates for azole resistance. Methods to be considered are agar-based resistance screening followed by MIC-testing if growth on azole-containing wells is observed. Where phenotypic screening takes 24–48 h, PCR directly on clinical samples can potentially detect *cyp51A* mediated resistance much quicker, provided sensitivity and coverage of azole resistance markers of the assay are sufficient, but non-*cyp51A* mediated resistance will not be detected [65]. Finally, it would be preferable to include imidazole drugs in in vitro susceptibility tests as they are used for treatment. However, clinical breakpoints will need to be established to interpret MIC values. Reconsidering the use of azole therapy as primary treatment for animals is impaired by lack of alternatives. In the European Union only imidazole and triazole antifungal drugs are registered for therapy in animals [55]. Fortunately, off-label AMB treatment has been described [1] and offers an alternative treatment option in animals with azole-resistant *Aspergillus* diseases.

Our study shows that in the Netherlands azole resistance is present in clinical *A. fumigatus* isolates from animals and at similar frequency as found in humans. We therefore advise veterinarians to be aware of azole resistance as cause of aspergillosis treatment failure and consider resistance screening of relevant isolates. The predominantly *cyp51A* TR-mediated resistance mutations support an environmental route of resistance selection, and warrants further studies from a One Health perspective.
